# Low-Energy Ion Implantation and Deep-Mesa Si-Avalanche Photodiodes with Improved Fabrication Process

**DOI:** 10.3390/s24020640

**Published:** 2024-01-19

**Authors:** Tiancai Wang, Hongling Peng, Peng Cao, Qiandong Zhuang, Jie Deng, Jian Chen, Wanhua Zheng

**Affiliations:** 1 Laboratory of Solid State Optoelectronics Information Technology, Institute of Semiconductors, Chinese Academy of Sciences, Beijing 100083, China; wangtiancai@semi.ac.cn (T.W.); hlpeng@semi.ac.cn (H.P.); pengkt11@semi.ac.cn (P.C.); 2 College of Electronic and Communication Engineering, University of Chinese Academy of Sciences, Beijing 101408, China; 3 State Key Laboratory on Integrated Optoelectronics, Institute of Semiconductors, Chinese Academy of Sciences, Beijing 100083, China; 4 Center of Materials Science and Optoelectronics Engineering, University of Chinese Academy of Sciences, Beijing 100049, China; 5 Physics Department, Lancaster University, Lancaster LA1 4YB, UK; q.zhuang@lancaster.ac.uk; 6 Southwest Institute of Technical Physics, Chengdu 610041, China; ariesshion@sina.cn (J.D.); gzuchenjian@126.com (J.C.)

**Keywords:** silicon avalanche photodiode, multiple epitaxy technology, ion implantation

## Abstract

Since the avalanche phenomenon was first found in bulk materials, avalanche photodiodes (APDs) have been exclusively investigated. Among the many devices that have been developed, silicon APDs stand out because of their low cost, performance stability, and compatibility with CMOS. However, the increasing industrial needs pose challenges for the fabrication cycle time and fabrication cost. In this work, we proposed an improved fabrication process for ultra-deep mesa-structured silicon APDs for photodetection in the visible and near-infrared wavelengths with improved performance and reduced costs. The improved process reduced the complexity through significantly reduced photolithography steps, e.g., half of the steps of the existing process. Additionally, single ion implantation was performed under low energy (lower than 30 keV) to further reduce the fabrication costs. Based on the improved ultra-concise process, a deep-mesa silicon APD with a 140 V breakdown voltage was obtained. The device exhibited a low capacitance of 500 fF, the measured rise time was 2.7 ns, and the reverse bias voltage was 55 V. Moreover, a high responsivity of 103 A/W@870 nm at 120 V was achieved, as well as a low dark current of 1 nA at punch-through voltage and a maximum gain exceeding 1000.

## 1. Introduction

Avalanche photodiodes (APDs) are widely used in various applications, such as medical imaging, sweeping robot guidance, light detection and ranging (LiDAR) [1], visible light communication [2,3], and single-photon detection [4,5,6], due to their internal amplification mechanism [7]. Compared to other types of sensors, silicon APDs work primarily by converting the incident light signal into an electrical signal and amplifying the current [8,9,10]. For near-IR wavelength applications (900~1100 nm), especially in weak light detection scenarios, silicon avalanche photodiodes are more popular compared to group III–V or II–VI compound semiconductor-based APDs and Ge/Si APDs [11,12], which is attributed to their low cost and compatibility with mature complementary metal–oxide–semiconductor (CMOS) technology.

Silicon APDs currently predominantly employ a planar structure based on bulk silicon substrate, and for the planar fabrication processes, guard ring structures are required to prevent premature breakdown (PBD) [13,14]. Based on these designs, in 2004, I. Wegrzecka et al. discussed and summarized the planar structure developed at the Institute of Electron Technology (ITE) [7]; these photodiodes were optimized for high gain, high detectivity, and low noise, but the fabrication process was highly redundant, involving no less than seven photolithography steps and four ion implantation cycles (including high-energy ion implantation). In 2010, based on the CMOS process line, Woo-Suk Sul et al. employed a shallow trench as a guard ring [13], which reduced the cycles of ion implantation and thermal annealing (TA) at the edge of the active area, and effectively improved the fill factor to 67.1%. P.N. Aruev et al. also developed and fabricated an APD for the recording of IR signals with a leading and trailing pulse edge shorter than 3 ns using planar silicon chemical vapor deposition (CVD) technology in 2019 [15]. This APD exhibited a sensitivity of 80~85 A/W at wavelengths ranging from 900 to 1010 nm, and a dark current of 1.5 nA. However, this study only involved the epitaxial growth of a single layer of intrinsic silicon; thus, subsequent multiple steps of ion implantation and TA were still required in the fabrication process. In 2022, Liu et al. proposed a novel structure that eliminates the requirements for wafer-thinning and the double side metallization process compared with most commercial silicon APD products [16]. The structure was also based on intrinsic silicon and utilized a separated absorption charge (SACM) design. The fabrication process involved multiple high-energy ion implantation steps. Despite achieving a low temperature coefficient of 0.0077 V/K, the responsivity was only 0.22 A/W at 905 nm. Besides these, it has been reported that some mesa devices with photon-trapping microholes (PMTH) [17,18,19,20,21,22,23,24], which enhance the quantum efficiency for absorption in the visible and near-infrared spectral regions, have also been developed. However, the thinness of the absorption layer in the epitaxial structure results in low responsivity in the near-infrared (850~1064 nm) spectral wavelengths compared with planar structure silicon APD.

To resolve the challenges in the fabrication steps, cost, and device’s overall performance, we proposed and fabricated an ultra-deep mesa-structure silicon APD with low-energy ion implantation, an ultra-concise fabrication process, and a quick optical pulse response. In this work, we demonstrate an improved fabrication process utilizing an ultra-deep mesa structure for silicon APDs targeting photodetection applications in the visible and near-infrared spectral wavelengths. The optimized process exhibits reduced complexity, with the number of photolithography and ion implantation steps reduced by at least half compared to most previously reported articles, to the best of our knowledge. Furthermore, low-energy ion implantation (below 30 keV) was implemented for the single ion implantation step. Based on this streamlined ultra-concise process, a deep-mesa silicon APD (DMSI-APD) with a 140 V breakdown voltage was fabricated. The resulting device displayed a low capacitance of 500 fF, consequently yielding a rapid optical pulse response time shorter than 2.7 ns. A high responsivity of 103 A/W@870 nm was also attained. Moreover, the dark current was suppressed below 1 nA at punch-through state and a maximum gain of over 1000 at 95% breakdown voltage was achieved.

## 2. Design and Fabrication

**Figure 1:**
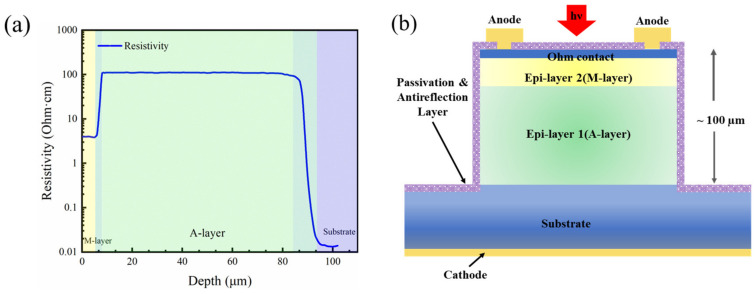
(**a**) Spreading resistance profile (SRP) of grown epitaxial wafers. (**b**) The structure cross-section of the ultra-deep mesa-structure silicon APD.

For the design epilayer of the silicon DMSI-APD presented in this work, the traditional separate absorption and multiplication (SAM) structure in the silicon APD is employed. However, unlike previously reported silicon APD fabrication processes, the main working layers in this paper were formed using multiple thick silicon CVD instead of ion implantation and a long-time thermal annealing drive in. Consequently, changes in the epilayer, including doping concentration and thickness, will influence the performance of the fabricated APDs, including dark current, breakdown voltage, and peak responsivity. In a previous study, we simulated the impact of the epi-absorption layer and the epi-multiplication layer on device performance [25]. The spreading resistance profile (SRP) measurement results of the epitaxial wafer are depicted in Figure 1a, while Figure 1b presents the cross-section schematic of the fabricated DMSI-APD. The device epilayer consists of an epi-absorption layer (A-layer) and a multiplication layer (M-layer), which are grown on a low-resistivity substrate using CVD technology. The A-layer and M-layer have a doping concentration and thickness of 1 × 10^14^ cm^−3^, 85 μm, 3.5 × 10^15^ cm^−3^, and 5 μm, respectively. All of these working layers were grown at 800~1000 °C. Furthermore, the thickness of the doping gradient layer from the substrate to the absorption layer and from the absorption layer to the multiplication layer is 10 µm and 2 µm, respectively, as shown in Figure 1a.

**Figure 2:**
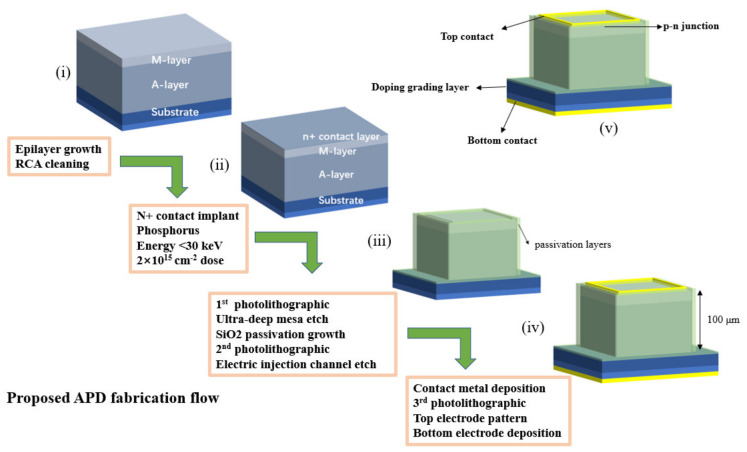
Fabrication process flow of the ultra-deep mesa silicon APD. The etched mesa sidewall was passivated with silicon dioxide (SiO_2_), which was grown through dry oxidizing followed by a wet oxidizing process at temperatures of 1050 °C and 600 °C, respectively. (i) Epitaxial wafer after growth; (ii) Wafer after phosphorus ion implantation; (iii) Wafer after mesa etch and oxide passivation; (iv) Wafer after metal deposition and lift-off; (v) Final fabricated device.

**Figure 3:**
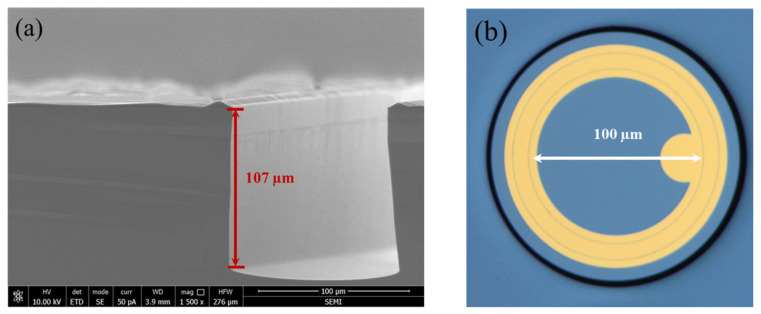
(**a**) Scanning electron microscope image of etched deep-mesa sidewall of the ultra-deep mesa-structure silicon APD. (**b**) Optical microscopy image of fabricated ultra-deep mesa-structure silicon APD.

To elucidate the fabrication process of the device more clearly, the improved ultra-concise process flow is shown in Figure 2. The fabrication consisted of four main steps. First, the epi-wafer was cleaned using buffered oxide etch to remove the natural oxide layer from the surface of the grown wafer (step (i) in Figure 2), followed by standard silicon wafer cleaning processes (organic reagents as well as strong acid reagents). Second, the p–n junction was formed through low-energy phosphorus implantation at 30 keV with a dose of 2 × 10^15^ cm^−2^ (step (ii) in Figure 2). Afterwards, the implanted dopants were activated and the implantation damage was repaired through rapid thermal annealing (RTA) with annealing parameters of 1050 °C for 60 s. Third, the first photolithography was performed and the ultra-deep mesa was etched using inductively coupled plasma (ICP) etching, resulting in a mesa depth of approximately 107 μm, as shown in Figure 3a. In addition, the etching gas was C_4_F_8_ and S_6_F (flow ratio 1:1.2) and the etching temperature was 180 °C, the ICP etch rate was around 0.5 μm/cycle, and a total of 200 cycles were performed in the etch process. Next, the dry etched mesa sidewalls were passivated with silicon dioxide (SiO_2_), dry-oxidized for 2 h, and wet-oxidized for 15 min at 1050 °C and 600 °C, respectively (step (iii) in Figure 2). Fifth, the second photolithography was completed and an electric injection channel was etched out, followed by the deposition and patterning of Ti/Au alloy to form the metal pads. Finally, the third photolithography was finished and another Ti/Au alloy contact was deposited after chemical–mechanical polishing (CMP) on the backside (step (iv) in Figure 2). Figure 2 (v) and Figure 3b show the three-dimensional (3D)-rendered illustration and the final optical microscopy image of the fabricated DMSI-APD, respectively. Figure 3a also shows the etch profile of the device, with an etch depth of about 107 μm for the mesa, from which it can be seen that the sidewall is about 90° steep, with a good etch sidewall roughness. The top table surface in Figure 3b is the active region of the device, and the inside of the metal ring is the photosensitive surface of the device. The shadows around the active region in Figure 3b are due to the significant drop in device height. Compared with other existing silicon APD fabrication processes [7], the proposed whole fabrication process of the improved DMSI-APD requires only three steps of standard contact UV lithography, and furthermore, the active region is formed with just one low-energy (<30 keV) ion implantation step and rapid thermal annealing. The method exhibits much potential for reducing the fabrication time and steps, and lowering the manufacturing costs, especially ion implantation costs.

## 3. Device Characterization and Discussion

### 3.1. I–V Characteristics and Responsivity

**Figure 4:**
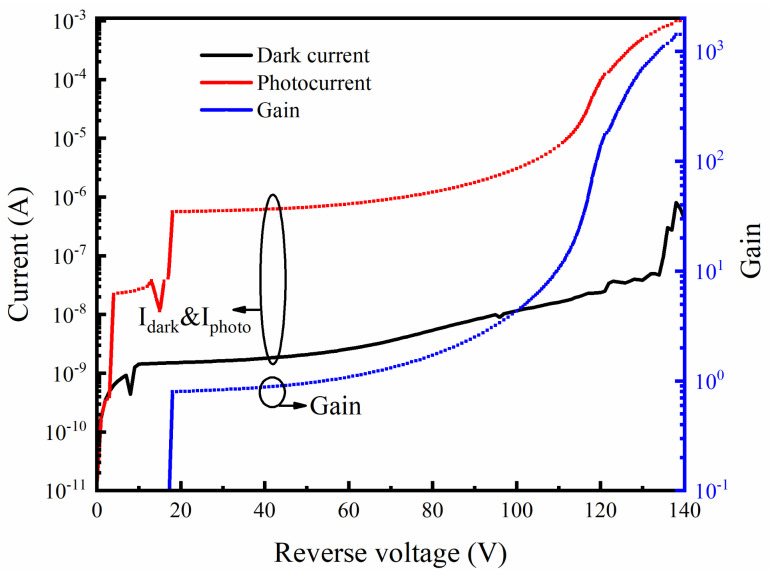
Reverse I–V characteristics and gain curve of DMSI-APD. The voltage step was set as 1 V and the Keithley noise current was around 50 pA.

Figure 4 shows the dark current and photocurrent of a typical fabricated DMSI-APD at room temperature. The I–V characteristics were obtained using a Keithley 2635B, with a voltage step of 1 V and a noise current of 50 pA. For the dark current measurement of the fabricated device, the DMSI-APD was placed in a dark box and data acquisition was accomplished simultaneously using the Keithley 2635B. For photocurrent measurement, the selected wavelength (905 nm) was illuminated perpendicular to the photosensitive surface of the device through an external optical circuit. To prevent damage to the device under test (DUT) due to the current overload caused by avalanche, the current compliance was set to 1 mA. Additionally, the calculated gain versus reverse voltage is shown in Figure 4, where the gain is defined by Equation (1):

(1) $$Gain=\frac{I_{photo}-I_{dark}}{I_{photo-punchthrough}-I_{dark-punchthrough}},$$

It can be observed that the dark current of the fabricated DMSI-APD device remains below 1 nA at the punch-through state. As the applied voltage to the device increases, the dark current shows a gradual increase until the reverse voltage reaches about 140 V. The breakdown voltage (Vbr) is defined as the voltage the dark current achieves at 10 µA [26], and for the fabricated device, the Vbr is approximately 140 V, which coincides with the previously designed structure value. In addition, the main reason why the dark current rises faster before the breakdown voltage is that, on the one hand, multiplication has already occurred inside the device, and, on the other hand, the passivation effect is not initially as desirable as it could be after a deeper etching depth.

**Figure 5:**
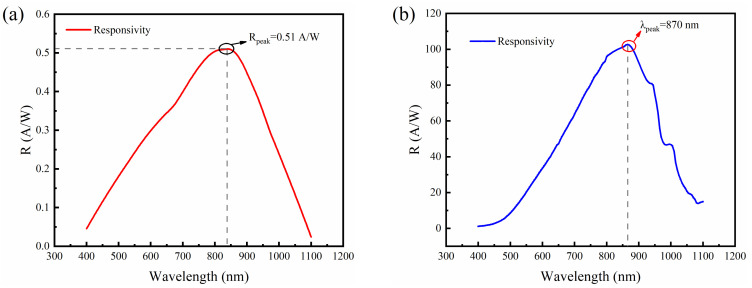
Measurement responsivity results of ultra-deep mesa-structure silicon APD at (**a**) 40 V; (**b**) 120 V.

In addition, from Figure 4, it can be seen that the maximum gain factor exceeds 1000 when the reverse voltage is near Vbr. Also, it is evident that as the device gain increases from 1 to 100, the corresponding voltage regulation range is 60 V (ranging from 50 V to 110 V), which indicates that the fabricated DMSI-APD has favorable linear dynamic performance characteristics. The favorable linearity of the DMSI-APD device makes it suitable for LiDAR dynamic sensing in linear mode. Based on Equation (2), the device responsivity under different applied voltages can also be calculated (shown in Figure 5). The responsivity is estimated according to Equation (2):

(2) $$R=\frac{I_{photo}(V)-I_{dark}(V)}{P_{in}},$$

where the parameter $P_{in}$ represents the total optical power entering the device at the selected wavelength.

Furthermore, the photo response spectrum of the device was tested by adjusting the monochromator so that the monochromatic light at different wavelengths of the white light source was irradiated to the photosensitive surface of the device, thus obtaining the response spectrum under the whole wavelength band. Figure 5 depicts the responsivity versus illumination wavelength of the DMSI-APD device at 40 V and 120 V. The fabricated device demonstrated a response wavelength range from 400 to 1100 nm. When the device was reverse-biased so that it was at unit gain, it achieved a peak responsivity of 0.51 A/W at 850 nm. By increasing the reverse bias voltage, the peak response wavelength experienced a red-shift to 870 nm, accompanied by a corresponding peak responsivity of about 103 A/W. Furthermore, it is worth noting that the responsivity of the fabricated DMSI-APD at 1100 nm remained at a significant value, exceeding 10 A/W, owing to the implementation of a meticulously designed antireflection (AR) coating in this study. To streamline the fabrication process, the AR coating was composed of silicon oxide with a specifically tailored thickness. In addition, since the present thermal oxidizer cannot control the growth thickness very accurately, resulting in the deviation of the final growth thickness from the design value, the growth thickness was subsequently optimized to move the peak response wavelength to the 905 nm wavelength.

### 3.2. Terminal Capacitance and Response Time

**Figure 6:**
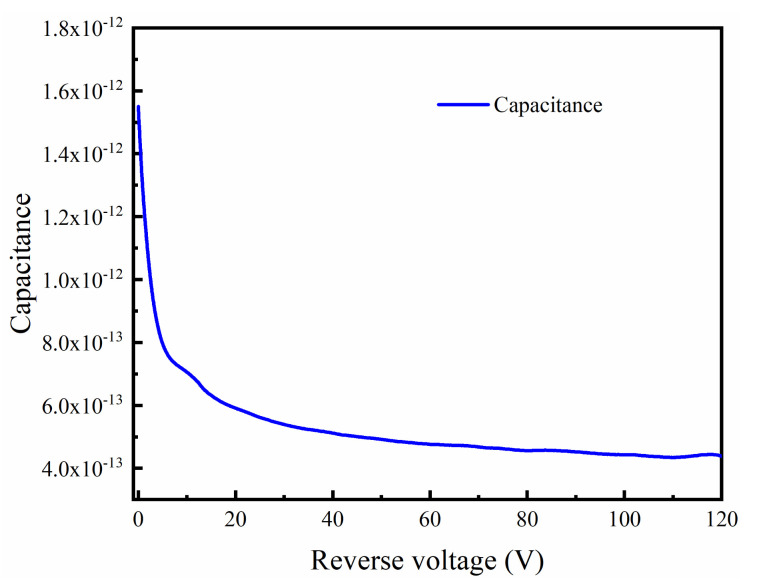
Capacitance versus reverse voltage of fabricated DMSI-APD.

The capacitance, as a function of reverse voltage, was measured and the rise time was calculated for the DMSI-APD device. Figure 6 illustrates the measured capacitance at various reverse voltages, with a measurement frequency of 1 MHz and a voltage step of 0.5 V. The results show a significant decrease in capacitance as the reverse voltage increased, and at the punch-through voltage state, the capacitance was below 500 fF, which was lower than that of most products, such as the Hamamatsu S14645 series. The main reason for the low capacitance is that the device depletion region is thicker and no additional parasitic capacitance was introduced after preparation, which also contributed to lowering the RC time constant of the device.

**Figure 7:**
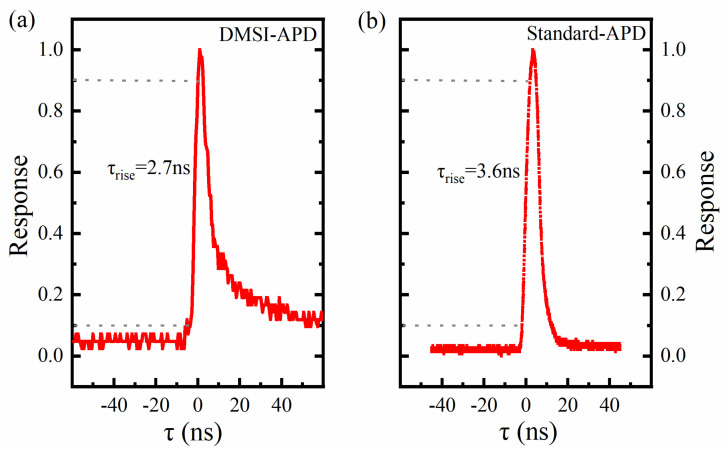
Dynamic characteristics for DMSI-APD: (**a**) quick optical pulse response of fabricated APD in this work; (**b**) quick optical pulse response of standard ET2020 APD.

Finally, to obtain the real response time and the calculated cut-off frequency of the DMSI-APD, the quick optical pulse response for the standard ET2020 APD (Standard APD) [27] and DMSI-APD were measured and calculated, as shown in Figure 7a,b. For the quick optical pulse response testing, a pulsed laser light source at 905 nm was used as the transmitter, the DMSI-APD was used as the receiver, and the signal from the detector response was output to an oscilloscope. It can be seen that the rise time of the DMSI-APD and standard APD were 2.7 ns and 3.6 ns at 55 V, respectively. Due to limitations in our test conditions, we were not able to perform measurements of the device rise time at different bias voltages, and plan to complete this work in the future.

## 4. Conclusions

In this work, an ultra-deep mesa-structure silicon APD with an improved concise fabrication process was proposed and fabricated. The improved process exhibited reduced complexity, with the number of photolithography and ion implantation steps being reduced by at least half of those involved in the existing process. Furthermore, only one low-energy ion implantation (below 30 keV) was implemented for the whole fabrication process. The fabricated ultra-deep mesa-structure silicon APD showed a 140 V breakdown voltage, a dark current below 1 nA, a maximum gain exceeding 1000, and a high responsivity of 103 A/W@870 nm. Moreover, the device has a low capacitance of 500 fF, consequently yielding a rapid optical pulse response time shorter than 2.7 ns at 55 V reverse voltage.

Limited by the sidewall roughness and defects introduced by the present etching, the dry oxidation time used was not long enough, which resulted in the defects and damage not being completely suppressed, thus causing the dark current of the device to fail to reach the desired value. In addition, due to the long etching time, the device also has high requirements for etching uniformity and repeatability, which are several optimizable points that we intend to continue to solve in our future work. After these issues are subsequently resolved, the proposed device will exhibit much potential for reducing the fabrication time and steps, and lowering the manufacturing costs, especially the ion implantation costs associated with weak light detection.

## Data Availability

The data that support the findings of this study are available from the corresponding author upon reasonable request.

## References

[1] Ahmad Z., Kuo S.-I., Chang Y.-C., Chao R.-L., Naseem N., Lee Y.-S., Hung Y.-J., Chen H.-M., Chen J., Goh C.S. (2022). Avalanche Photodiodes with Dual Multiplication Layers and Ultra-High Responsivity-Bandwidth Products for FMCW Lidar System Applications. IEEE J. Sel. Top. Quantum Electron..

[2] Tseng C.-K., Chen K.-H., Chen W.-T., Lee M.-C.M., Na N. (2014). A High-Speed and Low-Breakdown-Voltage Silicon Avalanche Photodetector. IEEE Photonics Technol. Lett..

[3] Lee M.-J. (2016). First CMOS Silicon Avalanche Photodetectors with Over 10-GHz Bandwidth. IEEE Photonics Technol. Lett..

[4] Van Sieleghem E., Süss A., Boulenc P., Lee J., Karve G., De Munck K., Cavaco C., Van Hoof C. (2021). A Near-Infrared Enhanced Silicon Single-Photon Avalanche Diode With a Spherically Uniform Electric Field Peak. IEEE Electron Device Lett..

[5] Dolatpoor Lakeh M., Kammerer J.-B., Aguenounon E., Issartel D., Schell J.-B., Rink S., Cathelin A., Calmon F., Uhring W. (2021). An Ultrafast Active Quenching Active Reset Circuit with 50% SPAD Afterpulsing Reduction in a 28 Nm FD-SOI CMOS Technology Using Body Biasing Technique. Sensors.

[6] Aull B.F., Schuette D.R., Young D.J., Craig D.M., Felton B.J., Warner K. (2015). A Study of Crosstalk in a $256 \times 256$ Photon Counting Imager Based on Silicon Geiger-Mode Avalanche Photodiodes. IEEE Sens. J..

[7] Wêgrzecka I., Wêgrzecki M., Grynglas M., Bar J., Uszyñski A., Grodecki R., Grabiec P., Krzemiñski S., Budzyñski T. (2004). Design and Properties of Silicon Avalanche Photodiodes. Opto-Electron. Rev..

[8] Hayati M., Majidifar S., Sobhani S.N. (2022). Using a Hybrid Encoding Method Based on the Hexagonal Resonators to Increase the Coding Capacity of Chipless RFID Tags. Int. J. RF Microw. Comput.-Aided Eng..

[9] Majidifar S., Karimi G. (2016). New Approach for Dielectric Constant Detection Using a Microstrip Sensor. Measurement.

[10] Pronko P.P., VanRompay P.A., Horvath C., Loesel F., Juhasz T., Liu X., Mourou G. (1998). Avalanche Ionization and Dielectric Breakdown in Silicon with Ultrafast Laser Pulses. Phys. Rev. B.

[11] Ishikawa Y., Wada K. (2010). Near-Infrared Ge Photodiodes for Si Photonics: Operation Frequency and an Approach for the Future. IEEE Photonics J..

[12] Zaimia R., Kaddour S., Mastour N., Baachaoui S., Jemai M., Ridene S., Raouafi N. (2023). Effect of Ni-Concentration on the Linear and Nonlinear Optical Properties of MoS _2_ Nanostructures. Int. J. Mod. Phys. B.

[13] Sul W.S., Oh J.H., Lee C.H., Cho G.S., Lee W.G., Kim S.D., Rhee J.K. (2010). Guard-Ring Structures for Silicon Photomultipliers. IEEE Electron Device Lett..

[14] Lee M.-J., Rucker H., Choi W.-Y. (2012). Effects of Guard-Ring Structures on the Performance of Silicon Avalanche Photodetectors Fabricated with Standard CMOS Technology. IEEE Electron Device Lett..

[15] Aruev P.N., Ber B.Y., Gorokhov A.N., Zabrodskii V.V., Kazantsev D.Y., Nikolaev A.V., Filimonov V.V., Shvarts M.Z., Sherstnev E.V. (2019). Characteristics of a Silicon Avalanche Photodiode for the Near-IR Spectral Range. Tech. Phys. Lett..

[16] Liu D., Li T., Tang B., Zhang P., Wang W., Liu M., Li Z. (2022). A Near-Infrared CMOS Silicon Avalanche Photodetector with Ultra-Low Temperature Coefficient of Breakdown Voltage. Micromachines.

[17] Rawat A., Ahamed A., Bartolo-Perez C., Mayet A.S., McPhillips L.N., Islam M.S. (2023). Design and Fabrication of High-Efficiency, Low-Power, and Low-Leakage Si-Avalanche Photodiodes for Low-Light Sensing. ACS Photonics.

[18] Jiang N., Zhang S., Jiang Y. (2023). A Novel Photodiode Array Structure with Double-Layer SiO_2_ Isolation. Semicond. Sci. Technol..

[19] Bartolo-Perez C., Chandiparsi S., Mayet A.S., Cansizoglu H., Gao Y., Qarony W., AhAmed A., Wang S.-Y., Cherry S.R., Saif Islam M. (2021). Avalanche Photodetectors with Photon Trapping Structures for Biomedical Imaging Applications. Opt. Express.

[20] Frey L., Marty M., André S., Moussy N. (2018). Enhancing Near-Infrared Photodetection Efficiency in SPAD With Silicon Surface Nanostructuration. IEEE J. Electron Devices Soc..

[21] Zang K., Jiang X., Huo Y., Ding X., Morea M., Chen X., Lu C.-Y., Ma J., Zhou M., Xia Z. (2017). Silicon Single-Photon Avalanche Diodes with Nano-Structured Light Trapping. Nat. Commun..

[22] Gao Y., Cansizoglu H., Ghandiparsi S., Bartolo-Perez C., Devine E.P., Yamada T., Elrefaie A.F., Wang S., Islam M.S. (2017). High Speed Surface Illuminated Si Photodiode Using Microstructured Holes for Absorption Enhancements at 900–1000 Nm Wavelength. ACS Photonics.

[23] Cansizoglu H., Bartolo-Perez C., Gao Y., Devine E.P., Ghandiparsi S., Polat K.G., Mamtaz H.H., Yamada T., Elrefaie A.F., Wang S.-Y. (2018). Surface-Illuminated Photon-Trapping High-Speed Ge-on-Si Photodiodes with Improved Efficiency up to 1700 Nm. Photonics Res..

[24] Gao Y., Cansizoglu H., Polat K.G., Ghandiparsi S., Kaya A., Mamtaz H.H., Mayet A.S., Wang Y., Zhang X., Yamada T. (2017). Photon-Trapping Microstructures Enable High-Speed High-Efficiency Silicon Photodiodes. Nat. Photonics.

[25] Wang T., Cao P., Peng H., Xu C., Song H., Zheng W. (2023). High-Uniformity 2 x 64 Silicon Avalanche Photodiode Arrays with Silicon Multiple Epitaxy Technology. Chin. Opt. Lett..

[26] Lee M.-J., Choi W.-Y. (2010). A Silicon Avalanche Photodetector Fabricated with Standard CMOS Technology with over 1 THz Gain-Bandwidth Product. Opt. Express.

[27] ET-2020SiPhotodetector. https://www.rayscience.com/product-14622.html.

